# Measurement and valuation of health providers’ time for the management of childhood pneumonia in rural Malawi: an empirical study

**DOI:** 10.1186/s12913-016-1573-5

**Published:** 2016-07-28

**Authors:** Fiammetta Maria Bozzani, Matthias Arnold, Timothy Colbourn, Norman Lufesi, Bejoy Nambiar, Gibson Masache, Jolene Skordis-Worrall

**Affiliations:** 1 Department of Global Health and Development, London School of Hygiene & Tropical Medicine, 15-17 Tavistock Place, London, WC1H 9SH UK; 2 Institute for Global Health, University College London, London, UK; 3 Munich Center of Health Sciences, Ludwig-Maximilians-Universität München, Munich, Germany; 4 Institute of Health Economics and Health Care Management, Helmholtz Zentrum München, Neuherberg, Germany; 5 ARI Programme, Ministry of Health, Lilongwe, Malawi; 6 Parent And Child Health Initiative (PACHI), Lilongwe, Malawi

**Keywords:** Time use, Pneumonia, Provider costs, Pneumococcal conjugate vaccine, PCV-13, Children, Malawi

## Abstract

**Background:**

Human resources are a major cost driver in childhood pneumonia case management. Introduction of 13-valent pneumococcal conjugate vaccine (PCV-13) in Malawi can lead to savings on staff time and salaries due to reductions in pneumonia cases requiring admission. Reliable estimates of human resource costs are vital for use in economic evaluations of PCV-13 introduction.

**Methods:**

Twenty-eight severe and twenty-four very severe pneumonia inpatients under the age of five were tracked from admission to discharge by paediatric ward staff using self-administered timesheets at Mchinji District Hospital between June and August 2012. All activities performed and the time spent on each activity were recorded. A monetary value was assigned to the time by allocating a corresponding percentage of the health workers’ salary. All costs are reported in 2012 US$.

**Results:**

A total of 1,017 entries, grouped according to 22 different activity labels, were recorded during the observation period. On average, 99 min (standard deviation, SD = 46) were spent on each admission: 93 (SD = 38) for severe and 106 (SD = 55) for very severe cases. Approximately 40 % of activities involved monitoring and stabilization, including administering non-drug therapies such as oxygen. A further 35 % of the time was spent on injecting antibiotics. Nurses provided 60 % of the total time spent on pneumonia admissions, clinicians 25 % and support staff 15 %. Human resource costs were approximately US$ 2 per bed-day and, on average, US$ 29.5 per severe pneumonia admission and US$ 37.7 per very severe admission.

**Conclusions:**

Self-reporting was successfully used in this context to generate reliable estimates of human resource time and costs of childhood pneumonia treatment. Assuming vaccine efficacy of 41 % and 90 % coverage, PCV-13 introduction in Malawi can save over US$ 2 million per year in staff costs alone.

## Background

Pneumonia is the leading cause of mortality in children under 5 years of age worldwide, causing an estimated 1.3 million deaths in 2011 [1]. The largest burden of morbidity and mortality is borne by the world’s poorest countries, with about half of all pneumonia deaths occurring in the African region [1]. In Malawi, a 13-valent pneumococcal conjugate vaccine (PCV-13) against *Streptococcus pneumoniae*, the pathogen responsible for the largest proportion of pneumonia cases, was introduced in 2011 with support from the GAVI Alliance.

The pace of new vaccine adoption has been accelerating in recent years, with more sophisticated and expensive products becoming increasingly available on the market [2]. This can put a strain on the Expanded Programme on Immunization (EPI), the World Health Organization’s (WHO) initiative aimed at securing access to vaccination for all children in developing countries. In order to achieve the Global Immunization Vision and Strategy advocated by the WHO and the United Nations Children’s Fund (UNICEF), the 72 poorest countries in the world will have increased their annual immunisation spending from approximately US$ 2.5 billion in 2005 to US$ 4 billion between 2006 and 2015 [3]. Since co-payments on vaccines and injection supplies procured are required from all countries seeking GAVI Alliance support to introduce a new vaccine [4], information on the cost-effectiveness of any additions to the EPI in low-income settings such as Malawi are vital for both the national Government, in deciding on uptake, and GAVI, in deciding whether to offer support. To inform these decisions, cost-effectiveness models compare the costs of introducing a vaccine with the cost of treating childhood pneumonia given the pre- and post-introduction incidence rates [5].

While the human resource (HR) costs of delivering a dose of vaccination have been previously estimated in a variety of high- and low-income settings [6–8], no studies to date have provided reliable and comprehensive treatment cost estimates in low-income countries that differentiate between in- and out-patient care, separate health provider and patient costs, and list the relative contributions of different cost components including HR [9–12]. The existing estimates of time spent by health care workers on specific conditions other than childhood pneumonia in sub-Saharan Africa suggest that there are substantial efficiency gains to be made by increasing their productivity [13, 14], and that the share of human resources costs represents approximately one third of total bed-day costs [15]. Reporting these estimates is particularly relevant given the ongoing human resource crisis in many parts of sub-Saharan Africa, including Malawi [16]. Treatment cost estimates for vaccine-preventable childhood illnesses are more prevalent in Latin America, although among the cost of illness studies included in a recent systematic review [17], only one reports the share of human resources costs, which was approximately 22 % of total under-5 pneumonia treatment costs [18].

Literature on the definitions and applications of existing methodologies for measuring and valuing the time of human resources for health is scarce and dated [19]. The ‘gold standard’ for this purpose is the time and motion technique, which has mostly been employed to analyse three sets of problems: adoption of new technologies, health workers productivity and tracking workload [20–22]. Other techniques in the literature include self-administered timesheets and patient interviews [19]. Data from time and motion studies are considered of better quality because of the use of external observers, who objectively record, label and time with a stopwatch all activities carried out by health facility staff throughout the working day. However, self-administered questionnaires were found to be in good agreement with time and motion results when measuring contact time with patients, as opposed to non-productive time [19], and were identified as a viable low-cost alternative to time and motion studies [23].

The objective of this study is to estimate the time spent by ward staff on case management activities for inpatients under the age of 5 diagnosed with severe and very severe pneumonia. We also aim to assign a monetary value to the health workers’ time, in order to produce an estimate of HR costs per childhood pneumonia admission and of the annual costs of childhood pneumonia both at the facility and at the national level. These results, together with existing estimates of the costs of delivering a dose of vaccine, can be used in economic evaluation to model the potential time and monetary savings resulting from the introduction of PCV-13 in Malawi. While the introduction is likely to have a positive cost in terms of staff time for training and vaccine administration, effective vaccination may reduce the severity of pneumonia episodes and, therefore, the number of pneumonia cases requiring admission. Moreover, reduced severity can lead to shorter hospital stays for those children who are admitted. In order to estimate the duration and costs of ward staff care, we collected time use data using self-administered timesheets alongside an ongoing full economic evaluation of PCV-13 introduction in Malawi (McCollum ED, Nambiar, B, et al. Impact of the 13-valent pneumococcal conjugate vaccine on clinical and hypoxemic childhood pneumonia at the community, health centre and hospital levels over three years in Malawi: a time series analysis of two districts, under review). The innovative use of this method in Malawi also enables us to reflect on the viability and usefulness of applying this method in a low-income context for calculating HR costs to be used in decision analytic modeling.

## Methods

### Data collection

Data was collected between June and August 2012 at Mchinji District Hospital, a secondary-level government facility in rural Malawi. We aimed to recruit a sample of 30 severe and 30 very severe pneumonia inpatients under the age of five from the paediatric ward after obtaining informed consent from their guardians. Pneumonia severity was classified according to the WHO guidelines based on clinical signs adapted for Malawi and following oxygen saturation thresholds for hypoxaemic pneumonia (normal: 96–100 %; severe: 90 < 95 % saturation; very severe: <90 % saturation) [24, 25]. The children were then tracked from admission to discharge by the ward personnel who attended to them. The personnel were asked to record all activities carried out on or for the patient and the time spent on the patient during each day of hospital stay. Elapsed time was measured using a wall clock and rounded to the closest minute. Only the time spent by clinical and support staff directly involved in care provision was measured. The time of overhead staff outside the admission ward that did not have direct contact with the children (i.e. kitchen, laundry, security, administration, maintenance and cleaning services staff) was not included.

Ward staff were trained to fill in the study forms and spot checks were performed by three study clinicians based at the facility on a daily basis to avoid over-reporting of the time spent with patients. To further reduce reporting bias, during training it was emphasized that the study forms were not collected for the purpose of appraising performance. In addition to regular oversight, spot checks were conducted by two external supervisors to monitor data quality and the handling of questionnaires, as well as to troubleshoot any issues arising during implementation. The data collection forms were structured as logbooks, as this was found to be the easiest format for facility staff to follow. One form was filled for each patient, where all activities and their duration on each day of admission were recorded. On each form, ward staff were also asked to record the admission and discharge dates, diagnosis and pneumonia severity as well as staff cadres. The complete list of activities and health worker cadres included in the study is shown in Table 1. An average of 8 h per shift and 5 shifts per week was used in the analysis, which corresponds to the official policy in Malawi. This was agreed upon in consultation with clinical staff as a reasonable average, taking into account both overtime and absences.

**Table 1:** Description of activities and staff involved in the management of under-5 pneumonia inpatients

Activities	Personnel	*N*
Assessment, monitoring and stabilising^a^	Senior clinical cadres	
OPD assessment/admission	Nursing officer	1
Monitoring oxygen saturation	Senior clinical technician	1
Oxygen therapy	Other clinical cadres	
Monitoring vital signs	Clinical technician	4
Intravenous fluid infusion	Nurse technician	12
Oral rehydration	Medical assistant	8
Administering injections	Auxiliary nurse	1
Benzylpenicillin	Health surveillance assistant	1
Chloramphenicol	Support staff	
Ceftriaxone	Hospital attendant	1
Gentamicin	Ward clerk	1
Administering other drugs		
Paracetamol		
Cotrimoxazole		
Salbutamol		
Aminophylline		
Quinine		
Artemisinin-based combination therapy		
Diagnostic tests		
HIV antibody test		
Malaria blood film		
Administrative tasks		
Ward round		
Discharge paperwork		
Other paperwork		
Setting review appointment		

^a^ during the study period, no x-ray facilities were available at Mchinji District Hospital

A monetary value was then assigned to the time spent on each patient by allocating a corresponding percentage of the health workers’ monthly salary. The official salary scales for all clinical and support staff were obtained from the Ministry of Health and median salaries for each cadre, which assumed a seniority level corresponding to 3.5 years of experience, were used in the calculations. All costs are presented in 2012 US$.

To move from the time spent on index or sample patients to an estimate of time spent treating all pneumonia cases presenting at health facilities in Malawi, we first multiplied the cost estimates by the number of patients with severe and very severe pneumonia admitted during 1 year. We then multiplied the annual human resource cost at the hospital by the annual rate of pneumonia cases in children under the age of five presenting at health facilities in the country. The time horizon of 1 year was selected as it is the most common budgeting period for health facilities. The total number of inpatients admitted to Mchinji District Hospital during 2012 was obtained from hospital surveillance data collected as part of the PCV-13 effectiveness trial taking place alongside the economic evaluation (McCollum ED, Nambiar, B, et al. Impact of the 13-valent pneumococcal conjugate vaccine on clinical and hypoxemic childhood pneumonia at the community, health centre and hospital levels over three years in Malawi: a time series analysis of two districts, under review). Estimates of the number of children under the age of five and of pneumonia incidence rates in this age group were obtained from the Comprehensive Multi-Year Plan (cMYP) submitted to the GAVI Alliance for the periods 2010–2014 and 2012–2016 [26, 27].

The study received ethics approval from the National Health Sciences Research Committee in Malawi (protocol 941) and from the University College London (UCL) Research Ethics Committee (protocol 2006/002).

### Data analysis

The data were analysed in Stata, Version 12. The mean and standard deviation of the minutes spent on different activities were calculated and stratified by pneumonia severity. When more than one activity was recorded in one time slot by the same individual staff member, a weight was applied to each activity based on its average duration when performed on its own. For example, when a member of staff reported monitoring vital signs during a ward round, the time assigned to monitoring vital signs was equal to the average duration of the activity when performed individually.

Differences in the duration of activities between severe and very severe cases were analysed by t-tests using individual activities rather than patients as the unit of analysis, to investigate whether the same activity took longer when performed for very severe than for severe cases.

After assigning a monetary value to the time spent on pneumonia admissions, the net time costs at population level were modeled using estimates of vaccine efficacy and pneumonia prevalence. Sensitivity analysis was performed by varying estimates of the reduction in the incidence of severe pneumonia after PCV-13 introduction. The vaccine efficacy estimates for PCV-13 of 65 % (range: 22–100 %) against all-cause very severe pneumonia and 0 % against all-cause severe pneumonia were based on the results of the PCV-13 effectiveness trial (McCollum ED, Nambiar, B, et al. Impact of the 13-valent pneumococcal conjugate vaccine on clinical and hypoxemic childhood pneumonia at the community, health centre and hospital levels over three years in Malawi: a time series analysis of two districts, under review). Vaccine coverage 3 years post-introduction was 75 % (McCollum ED, Nambiar, B, et al. Impact of the 13-valent pneumococcal conjugate vaccine on clinical and hypoxemic childhood pneumonia at the community, health centre and hospital levels over three years in Malawi: a time series analysis of two districts, under review). A positive and negative variation of 25 % in the annual number of pneumonia admissions under the age of five at Mchinji District Hospital was also considered in the sensitivity analysis.

## Results

Data were collected for 28 severe and 24 very severe pneumonia cases under the age of five. The caregivers of five children, one with severe and four with very severe pneumonia, refused to take part in the study. The target number of participants, 30 for each diagnosis, was not achieved due to a lower than expected number of cases presenting at the facility during the recruitment period. Nineteen participants were female and 33 were male. Mean age was 15 months, with 75 % of the children aged below 2 years. The average duration of hospital admission was 4 days for children with severe pneumonia (SD = 1.02) and 5 days for very severe cases (SD = 1.79).

**Fig. 1:**
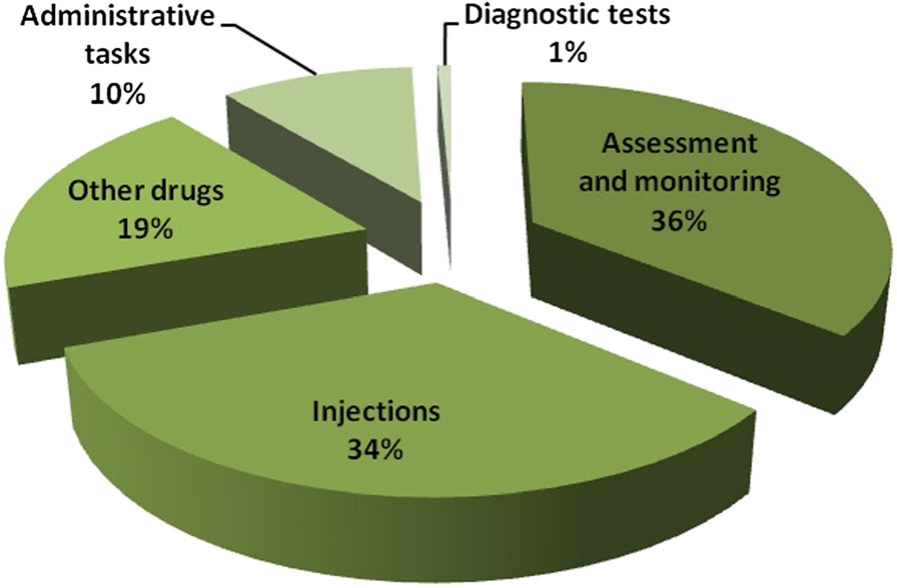
Description of reported activities by type (*n* = 1071)

A total of 1017 entries, grouped according to 22 different activity labels, were recorded during the observation period. On average, 99 min (SD = 46) were spent on care and case management activities for each admission: 93 min (SD = 38) for severe and 106 min (SD = 55) for very severe cases. Figure 1 shows the distribution of recorded activities by type: approximately 36 % of activities involved monitoring and stabilization of the child’s condition, including administration of non-drug therapies such as oxygen, oral rehydration and intravenous fluids, and a further 34 % of the time was spent on injecting antibiotics.

The average duration of individual activities per admission according to pneumonia severity is presented in Table 2. The most time-consuming activity for both severe and very severe cases was administering antibiotics by injection, which was performed an average of 3 times per day for each child. The mean time per admission for injecting benzylpenicillin, including preparation time, was 24 min for severe pneumonia cases (SD = 17 min) and 4 min for very severe cases (SD = 7 min), while injecting chloramphenicol took on average 2 min among severe cases (SD = 5 min) and 22 min (SD = 14 min) among very severe. No strong evidence of any single task lasting significantly longer for more or less severe patients was found except for oxygen therapy, which was administered to 5 out of 28, and 20 out of 24 children with severe and very severe pneumonia, respectively (*p* = 0.001, Table 2). While most activities were performed for both severe and very severe cases, antimalarials were administered to one in five severe cases and never to very severe cases, suggesting less uncertainty in the diagnosis of very severe pneumonia even in the absence of a functioning x-ray machine.

**Table 2:** Time spent on individual activities for severe (*n* = 28) and very severe inpatients (*n* = 24)

	Severe		Very severe		
	*N*	Mean (SD)	*N*	Mean (SD)	*P*-value*
Injecting antibiotics	241	25.78 (16.63)	42	29.16 (18.35)	0.24
Oral medications	177	15.21 (9.34)	159	16.42 (14.26)	0.36
OPD assessment	28	12.70 (9.93)	24	9.1 (8.45)	0.08
Monitoring oxygen saturation	21	7.25 (10.94)	67	9.71 (10.97)	0.21
Ward round	49	4.78 (4.04)	48	5.37 (7.60)	0.36
Oxygen therapy	5	0.36 (0.95)	20	4.87 (8.45)	0.001
Discharge/paperwork	45	3.36 (2.23)	50	2.71 (3.03)	0.50
Monitoring vital signs	12	1.25 (3.48)	10	1.46 (2.22)	0.40
Intra-venous fluid	3	0.32 (1.06)	3	1.79 (6.09)	0.11
Diagnostic tests	3	0.42 (1.26)	5	0.75 (1.73)	0.22
Administering antimalarials	5	0.28 (1.01)	0	0 (0)	0.08
TOTAL	589	93.14 (37.99)	428	106.43 (54.76)	0.15

*N* total observations for each activity, *OPD* out-patient department

Injecting antibiotics: benzylpenicillin, chloramphenicol, ceftriaxone, gentamicin

Oral medications: paracetamol, cotrimoxazole, salbutamol, aminophylline

Antimalarials: artemisinin-based combination therapy, quinine. Diagnostic tests: malaria blood film, HIV antibody test

* T-test for a difference in mean time spent on activity between severe and very severe cases

### Staff time

Nurses supplied 60 % of the total time spent on pneumonia admissions, other clinicians 25 % and support staff 15 %. Table 3 summarises the time per patient spent by senior, specialised and support personnel with severe and very severe cases. While there is no evidence of a significant difference in the amount of time provided by senior clinical staff and nurses between severe and very severe cases, support staff such as hospital attendants and ward clerks worked significantly longer on children with very severe pneumonia than on severe cases (*P* = 0.01).

**Table 3:** Human resources time and costs for severe and very severe pneumonia case management at Mchinji District Hospital

	All	Severe pneumonia	Very severe pneumonia	*P*-value
Mean time (SD), minutes				
Senior clinical cadres	3.26 (8.22)	2.93 (7.94)	3.67 (8.68)	0.37
Clinical cadres	73.27 (34.76)	72.29 (37.68)	74.42 (31.76)	0.41
Support staff	11.44 (14.98)	7.28 (7.27)	16.29 (19.75)	0.01
HR costs, US$				
Cost per child	30.99	29.52	32.69	
Annual costs	24,947	14,817	9,906	
One-way sensitivity analysis				
Annual cost savings (range)	6,439			
^a^	(2,179–9,906)			
^b^	(−978–11,495)			

^a^ Based on PCV-13 vaccine efficacy estimates of 65 % (range: 22–100 %) against all-cause very severe pneumonia and 0 % against all-cause severe pneumonia (McCollum ED, Nambiar, B, et al. Impact of the 13-valent pneumococcal conjugate vaccine on clinical and hypoxemic childhood pneumonia at the community, health centre and hospital levels over three years in Malawi: a time series analysis of two districts, under review)

^b^ Based on a +/− 25 % increase in the annual number of pneumonia inpatients under five years of age

Based on the average length of hospital stay for children in the sample, nurses spent an average of 12 min per bed-day on children with pneumonia (2 % of their working hours), senior clinicians spent 5 min per bed-day (1 %) and support staff spent 2 min per bed-day (0.4 %). Senior clinical staff were in contact with the patients mainly to prescribe and administer drug regimens (38 % of average time spent per admission) and during ward rounds (26 %), with the remaining time dedicated to administrative activities. Nurses and clinical technicians were also involved with administering antibiotics and other drug regimens (58 % of average time spent per admission) and most of the remaining contact time with patients came during the OPD assessment and admission (15 %) and monitoring of oxygen saturation and vital signs (13 %). Support staff were mostly involved in administering drugs (57 % of average time spent per admission), assisting clinicians with the paperwork (20 %) and during ward rounds (14 %).

### Costs of human resources

#### Mchinji District Hospital

In 2012, 303 children under 5 years of age with very severe pneumonia and 502 with severe pneumonia were admitted to Mchinji District Hospital. Using median salaries for each cadre and the average exchange rate for 2012 (US$ 1 = MWK 248.59), the cost of salaries per bed-day was US$ 2.02 for severe and US$ 2.20 for very severe pneumonia cases. Table 3 shows costs per child based on the average length of hospital stay in our sample. At an HR cost per child of US$ 31, Mchinji District Hospital spent approximately US$ 24,947 on pneumonia inpatients under the age of five in 2012. Based on vaccine efficacy estimates of 65 %, the facility could save around US$ 6,439 in staff salaries every year due to the reduction in under-five pneumonia cases brought about by the new vaccine.

#### National estimates

According to cMYP projections, 2,672,068 children under the age of five lived in Malawi in 2012 [27]; and the rate of pneumonia cases presenting at health facilities in the same age group was 4,212/100,000 child years [26]. This translates to annual HR costs of childhood pneumonia case management of US$ 3,487,875. Reductions in incidence due to PCV-13 introduction would free up an estimated US$ 2,057,846 (range: US$ 1,709,059–US$ 2,406,634) every year, that the Malawian Government currently spends on the ward staff caring for pneumonia inpatients under the age of five.

## Discussion

This study provides estimates of the time spent by ward staff on all activities related to pneumonia case management in children under five in a rural district hospital in Malawi. Monetary valuations of the time are provided in order to estimate the potential savings from reductions in the number of pneumonia cases presenting at health facilities after the introduction of PCV-13. According to our estimates, at an average HR cost of around US$ 2 per bed-day, Mchinji District Hospital spends approximately US$ 24,947 every year in staff salaries alone. About one third of this could be freed up for alternative use if the projected reductions in the burden of childhood pneumonia are realised. The estimated savings of over 2 million US$ at the national level would also be substantial, considering that this constitutes roughly 0.8 % of total health expenditure for Malawi in 2012 [28].

There are few comparable estimates of time and cost of HR in the literature: this is the only known time use study conducted in Malawi and among the first from a low- or middle-income setting [6, 13, 14]. None of the available results obtained using a similar method covered under-5 pneumonia case management. Existing estimates of the HR costs of pneumonia treatment from cost-effectiveness studies of PCV-13 introduction used different methodologies with lower levels of accuracy: of the three available economic evaluations of PCV-13 from sub-Saharan Africa, two utilised treatment cost estimates, including HR costs, from secondary data sources [11, 12], and one reported societal cost estimates without differentiating between health provider and patient costs, so that it is not possible to identify the HR component [29]. Chola and Robberstad reported provider costs of pneumonia of US$ 48 per outpatient visit and US$ 215 per admission in Zambia, although no breakdown of the individual cost components, such as HR, is presented [10]. The costs of childhood pneumonia were considerably higher than those reported for under-five diarrhoea in the same study, which were US$ 26 for outpatient visits and US$ 78 per bed-day, respectively [10]. Only one study from Fiji provided comprehensive provider cost estimates for childhood pneumonia of US$ 19 per outpatient visit, with personnel costs accounting for approximately 90 % of the cost [30]. However, this estimate cannot be reliably compared to our results as it includes the costs of pharmacy and overhead staff and a corresponding estimate for inpatients is not reported.

Use of self-administered timesheets allowed us to analyse the differences in both the mix of activities and the types of human resources involved in case management between severe and very severe pneumonia cases. We found that the excess contact time with very severe cases was mostly provided by non-clinical support staff, such as hospital attendants, whose main tasks involved administering drugs and assisting with paperwork. This finding is of relevance empirically, as we show that the difference in staff time allocation and cost between severe and very severe pneumonia case management is mostly driven by support staff in low-income settings. It is also an important methodological finding, as this is the first study to demonstrate that self-administered timesheets can be used effectively among support staff in these settings.

In terms of differences in treatment options, approximately one in 5 severe cases received antimalarials, while they were not prescribed to any of the very severe cases. This suggests that there may be some uncertainty in the diagnosis when symptoms are not very severe and that malaria is being treated as a ‘fail-safe’ diagnosis. An unintended consequence of vaccine introduction might therefore be that the number of misdiagnosed cases of malaria and the unnecessary prescription of antimalarials may increase.

It should be noted that, while self-administered timesheets had significant advantages in this context, the study had some limitations. Firstly, the number of tracked patients is relatively small and the study was conducted at one single facility. While we have assumed, in the absence of evidence to the contrary, that the findings from this study population can be generalised to the national level, this level of generalisation should be treated with caution. In addition, at the time this study was conducted, Mchinji District Hospital did not have a functional x-ray unit, thus potentially depriving clinical staff of an important tool for diagnosing pneumonia. This is, however, a common occurrence in resource-poor settings and we believe that our results are informative and applicable to the majority of secondary hospitals in Malawi and other countries in the region. Another limitation of the present study is that no reliable estimates of herd immunity and of potential reductions in incidence of adult pneumonia due to vaccination were available. The estimated savings are therefore likely to be underestimated and conservative. However, this effect could be balanced out by the potential increase in incidence due to serotype replacement and the appearance of other pathogens. The vaccine effectiveness trial, moreover, found a significant increase (58 %, range: 22–96 %) in non-severe cases which, although less deadly, will result in additional costs to health providers (McCollum ED, Nambiar, B, et al. Impact of the 13-valent pneumococcal conjugate vaccine on clinical and hypoxemic childhood pneumonia at the community, health centre and hospital levels over three years in Malawi: a time series analysis of two districts, under review). These additional costs will be particularly felt at the primary care level and by community health workers following the revision of the WHO Integrated Management of Childhood Illness guidelines, which now recommend to treat some of the cases previously classified as severe pneumonia with oral amoxicillin and home care [31]. We therefore believe that these estimates of the HR costs of pneumonia treatment are the most accurate available and a powerful tool for advocacy in favor of mobilising funds in support of immunisation.

In terms of practical feasibility, self-administered timesheets monitored by internal observers were found to be relatively inexpensive and effective for collecting time-use data at Mchinji District Hospital. Key characteristics of the study facility that made the technique suitable in this context include the physical size of the children’s ward, which occupies a single large room, and the relatively small number of staff working on the ward (approximately 30 people at any one time). A single room is easier to monitor and a smaller number of employees is quicker and cheaper to train. In our experience, the use of a data entry form structured like a logbook was easy to handle by ward staff, who did not perceive data entry to be unduly adding onto their daily workload. Since the timesheets are comparatively more straightforward than other data collection methods, the clinical staff on the ward readily bought into the study and agreed to be recruited as day-to-day supervisors, so that data quality was ensured without having external observers on site on a daily basis. However, since self-completion forms rely heavily on the participation of facility staff in the planning phase, we would not recommend this technique in larger facilities, where fluctuations in ward personnel are usually higher, study preparation times might be longer and inclusion of a larger number of supervisory staff from other specialties, such as radiologists, might be necessary. In such a setting, where increasing the number of facility staff and the amount of their time on the study may be less feasible, it might be best to hire external enumerators and supervisors and carry out a standard time and motion study, thus likely increasing overall costs.

## Conclusions

This study makes innovative use of an established method to estimate and value the time spent on case management of childhood pneumonia in Malawi. Based on the number of pneumonia inpatients admitted at the study facility during 2012 and available vaccine efficacy estimates, immunisation could result in over US$ 2 million annual cost savings on staff salaries alone. We have demonstrated that self-administered timesheets can be cheaply and successfully used in this setting to measure the time spent by clinical and support staff on different activities. These measures, in turn, can be used to generate reliable estimates of the HR cost savings following PCV-13 introduction in Malawi.

## Abbreviations

cMYP, Comprehensive Multi-Year Plan; EPI, expanded Programme on Immunisation; GAVI, GAVI Alliance; HR, human resource; MWK, Malawian Kwacha; OPD, outpatient department; PCV-13, 13-valent pneumococcal conjugate vaccine; SD, standard deviation; UCL, University College London; UNICEF, United Nations’ Children Fund; WHO, World Health Organization
